# Mitogen‐activated protein kinase kinase kinase 1 facilitates the temozolomide resistance and migration of GBM via the MEK/ERK signalling

**DOI:** 10.1111/jcmm.70173

**Published:** 2024-10-23

**Authors:** Sicheng Wu, Senrui Xue, Yuchen Tang, Wenyu Zhao, Maojin Zheng, Zhixuan Cheng, Xin Hu, Jinmin Sun, Jing Ren

**Affiliations:** ^1^ Jiangsu Key Laboratory of Brain Disease Bioinformation, Research Center for Biochemistry and Molecular Biology Xuzhou Medical University Xuzhou Jiangsu China; ^2^ School of Life Sciences Xuzhou Medical University Xuzhou Jiangsu China; ^3^ Laboratory of Clinical and Experimental Pathology, Department of Pathology Xuzhou Medical University Xuzhou Jiangsu China

**Keywords:** glioma, MAP3K1, MEK/ERK, migration, patients‐derived organoids, TMZ resistance

## Abstract

Mitogen‐Activated Protein Kinase Kinase Kinase 1 (MAP3K1) is overexpressed in gliomas; however, its clinical significance, biological functions, and underlying molecular mechanisms remain unclear. Abnormal overexpression of MAP3K1 in glioma is strongly associated with unfavourable clinicopathological characteristics and disease progression. MAP3K1 could potentially serve as a reliable diagnostic and prognostic biomarker for glioma. MAP3K1 silencing suppressed the migration but had no effect on the proliferation and cell death of Glioblastoma Multiforme (GBM) cells. MAP3K1 knockdown exacerbated the temozolomide (TMZ) induced inhibition of glioma cell proliferation and death of GBM cells. In addition, MAP3K1 knockdown combined with TMZ treatment significantly inhibited the growth and increased cell death in organoids derived from GBM patients. MAP3K1 knockdown reversed TMZ resistance of GBM in intracranial glioma model. In terms of molecular mechanisms, the phosphorylation level of ERK was significantly decreased by MAP3K1 silencing. No significant change in the JNK pathway was found in MAP3K1‐silenced GBM cells. Inhibition of ERK phosphorylation suppressed the migration and enhanced the TMZ sensibility of GBM cells. MAP3K1 was correlated with the immune infiltration in glioma. MAP3K1 could facilitate the migration and TMZ resistance of GBM cells through MEK/ERK signalling.

## 1 INTRODUCTION

Glioma is recognized as the central nervous system's most frequent and lethal primary malignant brain tumour.^1^, ^2^ Within this diverse group of tumours, it is believed that they originate from neuroglial stem or progenitor cells. Histologically speaking, adult‐type diffuse gliomas are classified into astrocytomas, oligodendrogliomas and glioblastomas based on their resemblance to the neuroglial cells found in the healthy brain.^3^, ^4^ Currently, the conventional clinical strategy for treating glioma involves maximal surgical resection, radiotherapy and TMZ chemotherapy, but it may not yield an ideal outcome for all patients.^5^ Additionally, recurrence and poor prognosis are attributed to the immunosuppressive environment and restricted delivery of chemotherapy or immunotherapy due to the blood brain barrier.^6^ At present, TMZ is the only first‐line chemotherapy drug for GBM treatment, and its drug resistance is widespread and increasingly prominent, which greatly limits the clinical efficacy of TMZ.^7^, ^8^, ^9^ Notably, the tremendous efforts made to research traditional therapies in these years have been in vain. Consequently, it is crucial to thoroughly examine potential molecular markers for diagnosis and prognosis, identify key molecules mediating TMZ resistance in GBM patients, and illuminate the molecular mechanisms underlying glioma.

MAP3K1, a serine–threonine kinase, is a member of the large MAP3K superfamily. It is the only member that contains multiple functional domains, including PHD, SWIM and RING motifs, and features of caspase cleavage sites and E3 ligase activity among the members of the MAP3K family.^10^ A variety of stimuli, including growth factors, cytokines, hormones and cell stresses such as oxidative stress, could activate MAP3K1. MAP3K1 is involved in the regulation of downstream signals, such as NF‐kappa‐B, JNK and p38 pathways, to determine the fate of cells.^11^, ^12^ Genetic analyses have provided insights into the crucial role of MAP3K1 in various biological processes (BP), including the immune system, wound healing, and tumorigenesis.^13^, ^14^, ^15^

MAP3K1 was identified as a causative gene by searching for the susceptibility alleles of breast cancer.^16^ MAP3K1 deficiency sped up melanomagenesis in mice and displayed a similar role in humans.^17^ In pancreatic cancer, MAP3K1 is overexpressed and inhibiting MAP3K1 activity effectively suppresses the metastasis of pancreatic cancer cells.^18^, ^19^ In prostate cancer, miRNA‐627 regulated the proliferation and apoptosis of prostate cancer cells through MAP3K1.^20^ While previous findings have suggested that MAP3K1 participates in the development of various tumour types, its precise role and gene expression pattern in glioma remain unclear. Here, we discovered that aberrantly expressed MAP3K1 was significantly associated with unfavourable clinicopathological characteristics and disease progression. MAP3K1 might be a promising diagnostic and prognostic biomarker for glioma. MAP3K1 was correlated with the immune infiltration in glioma. MAP3K1 silencing suppressed GBM cell migration and had no effect on the proliferation and death of GBM cells. Meanwhile, MAP3K1 knockdown reverse TMZ resistance of GBM in intracranial glioma model and GBM patients‐derived organoids. In addition, MAP3K1 might promote the TMZ resistance and migration of GBM cells through MEK/ERK signalling.

## 2 METHODS

### 2.1 Glioma tissues

Ninety‐six glioma tissue samples between 2016 and 2017 were collected from the Department of Pathology in the Affiliated Hospital of Xuzhou Medical University. Pathologists assessed these samples according to the 2021 World Health Organization (WHO) classification criteria. The study was undertaken with the understanding and written consent of each subject and conformed to the standards set by the Declaration of Helsinki. We obtained approval from the Institutional Ethics Committee of Affiliated Hospital of Xuzhou Medical University to ensure compliance with ethical standards for conducting this study.

### 2.2 Data acquisition

We acquired the relevant RNA‐seq data of gliomas from three databases: The Cancer Genome Atlas (TCGA) database (*n* = 703), the Chinese Glioma Genome Atlas (CGGA) database (batch I, *n* = 413; batch II, *n* = 273), and Genotype‐Tissue Expression (GTEx, *n* = 1157). To analyse the overall survival (OS) and MAP3K1 expression in glioma tissues of patients, we excluded defective data which didn't include complete clinical information and used 413 glioma tissues from dataset batch I and 273 glioma tissues from dataset batch II of the CGGA. The Gene Expression Profiling Interactive Analysis web server (http://gepia.cancer‐pku.cn/detail.php) was employed to examine the gene expression profiles in all tumour tissues.

### 2.3 Cell culture and transfection

Cell lines including U251, LN229, U87, U118 and T98G were obtained from the American Type Culture Collection. These cell lines were cultivated in Dulbecco's modified Eagle's medium (DMEM) supplemented with 10% fetal bovine serum, which was obtained from Keygen Biotech in Nanjing, China. The living environment of these cells was 37°C within a humid atmosphere containing 5% CO_2_. All cell lines have been authenticated and experiments were performed with mycoplasma‐free cells. All cells were periodically undergoing short tandem repeat profiling and verified in STR database to authenticate their identity every 1–2 years.

The MEK inhibitor U0126 was acquired from Beyotime (Shanghai, China). Both the MAP3K1 siRNA and scramble sequence were ourced from Ribobio in Guangzhou, China. Lipofectamine 3000 (L3000001, Invitrogen, America) was used for transfection according to the provided protocol. The sequences of siRNAs targeting MAP3K1 were siMAP3K1–1, gccacagtttagcggaaagaa; siMAP3K1–2, cctctcctttatctcatcatt; siMAP3K1–3, gcctttcgtatctccatgaaa. Efficiency of MAP3K1 knockdown was evaluated by Q‐PCR and immunoblot.

### 2.4 Plasmid construction

The MAP3K1 expression plasmids with same sense mutation of MAP3K1 coding sequences to avoid the degradation of the siMAP3K1–2 were designed and constructed by Gene Pharma Company (Shanghai, China). The sequences of siRNAs targeting MAP3K1 were siMAP3K1–2, cctctcctttatctcatcatt. The MAP3K1 coding sequence after same sense mutation was showed in Supplementary materials—Data S1 and cloned into the pcDNA3.1 plasmid.

### 2.5 Transwell assay

Eight micrometre transwell chambers (BD Biosciences, San Jose, CA) were used for transwell assays as previously described.^21^ Briefly, a cell suspension (1 × 10^5^ cells/well) in DMEM with 2% FBS was seeded into the upper chamber. The lower compartment of the Transwell chamber was added by DMEM with 10% FBS. Then, the remaining cells on the upper side of the chamber were removed, while the migrated cells were fixed with 4% PFA and stained with crystal violet. The stained cells were photographed and counted from five random fields.

### 2.6 Immunohistochemistry

Tissue blocks fixed in 4% paraformaldehyde and embedded in paraffin were sectioned into 4 μm slices using a microtome and incubated at 65°C for 2 h. The sections were deparaffinized in xylene and rehydrated with ethanol (100%, 80%, 70%). Antigen retrieval was performed by autoclaving in boiled sodium citrate buffer (pH 6). The sections were then treated with 3% hydrogen peroxide, blocked with 10% normal goat serum, and incubated overnight with anti‐MAP3K1 antibody (1:400, Santa Cruz, CA, United States). Horseradish peroxidase‐labelled secondary antibodies were applied, followed by DAB incubation and haematoxylin staining. The sections were dehydrated using ethanol (80%, 95%, 100%) and mounted with coverslips.

### 2.7 Western blotting

Harvested cells were lysed in RIPA buffer with protease inhibitors (Roche, Germany), and protein concentration was measured using the BCA Protein Assay Kit (Beyotime, Shanghai, China). The protein samples were subjected to polyacrylamide gel electrophoresis and transferred onto nitrocellulose membranes (Millipore). The membrane was blocked with bovine serum albumin and incubated overnight with primary antibodies against MAP3K1 (1:750; Santa Cruz, CA, United States), β‐actin (1:10000; ProteinTech, Rosemount, IL, Unitedstates) and GAPDH (1:10,000; ProteinTech, Rosemont, IL, United States). A horseradish peroxidase‐conjugated secondary antibody was then applied, followed by detection of proteins using enhanced chemiluminescence reagent.

### 2.8 Bioinformatics analysis

All the method of bioinformatics analysis (univariate and multivariate cox analyses, risk model constitution, single‐cell RNA sequencing analyses and function and pathway enrichment) was shown in Supporting information—Data S1.

### 2.9 Flow cytometry

Cell cycle staining kit bought from Keygen Biotech was employed following the manufacturer's protocols. Briefly, the collected cells were washed and suspended in a binding buffer. Then, cells were fixed with 70% cold dehydrated alcohol overnight and stained with PI. Cell death assay was performed by annexin‐V apoptosis detection kit (Keygen Biotech) according to the manufacturer's instructions. Flow cytometry analysed the samples immediately (FACSCanto II, Germany).

### 2.10 Cell viability

GBM cells were seeded in wells (5000/well), and their viability was assessed using the CCK8 kit (Vicmed, VC5001) after 24, 48, and 96 h in accordance with the protocol. The absorbance value of A450 was measured using a microplate reader.

### 2.11 Establishment and culture of GBM patients‐derived organoids

The fresh tumour tissues of GBM patients after surgery were collected in the operating room, put into rapid tissue preservation solution and transported on ice. Histopathological diagnosis of postoperative tumour tissues was performed, and organoids were established after GBM was confirmed. Y‐27632 (10 uM) should be added to the tissue preservation solution. The selected optimal area tumour tissue was cleaned several times and transferred to an anatomical microscope in a sterile laminar flow biosafety cabinet. The tissue was placed in a sterile glass dissecting dish and corneal scissors were used to cut into tissue blocks (0.5–1 mm). After cleaning, the best tissue blocks were selected by natural sedimentation method (bottom sinking within 30s), and the red blood cells were lysed and cleaned. The tissue blocks were placed in a 6‐well plate with ultra‐low adhesion, and GBM special organoid medium was added (Shanghai Lisheng Biotechnology). The culture plates were incubated in a CO_2_‐resistant shaker (120 rpm) at 37°C with 5% CO_2_ and 90% relative humidity, with medium changes every three and a half days. After successful culture, organoids underwent STR analysis and mycoplasma detection, followed by photography and histopathological examination.

### 2.12 3D live‐dead cell viability assay

MAP3K1 was knockdown by lentivirus infection in GBM patients‐derived organoid and treated by TMZ. The growth and cell death of GBM patients‐derived organoids were detected immediately after 3 weeks of TMZ treatment. Organoids during TMZ were tested using a Live‐Dead Cell Viability Kit (Sigma). Six millilitres cell medium was mixed with 6 mL PBS in a 1:1 ratio. A 12 mL dye solution was prepared by adding 5 μL calcein, 20 μL Propidium Iodide (PI) and 8 μL Hoechst 33342 to this mixture. The cell medium is aspirated from the culture plate and an appropriate amount of dye mixture is added to each well. Incubate at 37°C for 60 min. Fluorescence microscope was used to observe and photograph.

### 2.13 Glioma xenografts and in vivo imaging system

U87 cells with stable MAP3K1 knockdown and a negative control were infected with lentiviruses containing luciferase, and cell lines with stable luciferase expression were selected. For the glioma orthotopic xenotransplantation model, 5 × 10^5^ luciferase‐labelled U87 cells were stereotactically implanted into the right frontal cortex of 5‐week‐old female nude mice. Mice were anaesthetised, a midline incision was made, and a hole was drilled 1 mm lateral and 2 mm posterior to the bregma. Cells were injected in a 5 μL volume, 3 mm deep into the skull over 5 min. Temozolomide (12.5 mg/kg) was administered intraperitoneally on Day 7 post‐operation. Tumour growth was monitored in vivo using the Night OWL II LB 983 bioluminescent imaging system (Berthold Technologies, Germany). Animal procedures complied with the 3R rule and were approved by the Laboratory Animal Ethics Committee of Xuzhou Medical University.

### 2.14 Statistical analyses

The statistical analyses were conducted by utilizing GraphPad Prism 7.0 (GraphPad Software, Inc., San Diego, CA, United States) and SPSS16.0 software (SPSS 16.0; SPSS, Inc., Chicago, IL, United States). R (v.3.6.3) was utilized to merge statistical data acquired from the TCGA database. Correlation analyses were performed using the chi‐square (*χ* ^2^) test, Pearson's correlation, or Spearman's correlation analysis. Survival data from the CGGA database were analysed using the Kaplan–Meier method. The statistical significance of differences between groups was assessed with Student's *t*‐test. For comparisons involving more than two groups, one‐way ANOVA and the Kruskal–Wallis test were utilized. A *p*‐value of less than 0.05 was considered statistically significant (*p < 0.05, **p < 0.01, ***p < 0.001, ****p < 0.0001).

## 3 RESULTS

### 3.1 MAP3K1 is overexpressed and significantly associated with poor clinicopathological characteristics of glioma

**FIGURE 1:**
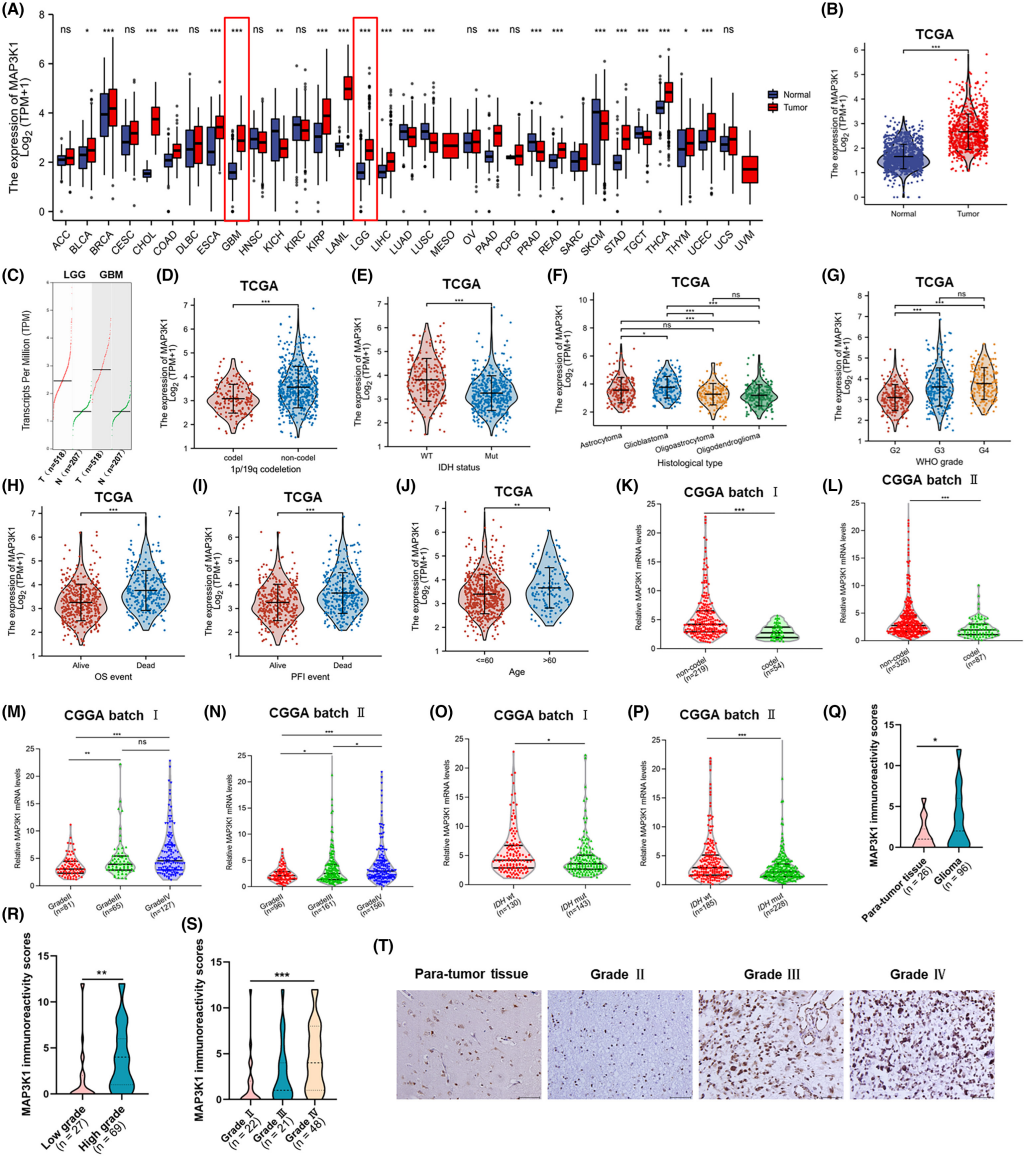
Aberrantly expressed MAP3K1 was significantly associated with poor clinicopathological characters of glioma. (A) Comparison of MAP3K1 expression between 33 types of tumours and normal samples from TCGA and Genotype‐Tissue Expression (GTEx) databases. (B) The mRNA expression of MAP3K1 in glioma and normal tissues from TCGA database and GTEx. (C) The transcript levels of MAP3K1 in glioma and corresponding normal tissues from the Gene Expression Profiling Interactive Analysis (GEPIA) web server. T, tumour tissue; N, normal tissue. The expression levels of MAP3K1 in different 1p/19q statuses (D), isocitrate dehydrogenase (*IDH*) genotypes (E), histological types (F) and WHO grades (G) of glioma analysed by TCGA database. The expression of MAP3K1 in different groups in view of the prognosis of glioma patients, including the OS (H), and PFI (I) event of glioma patients from TCGA. (J) The comparison of MAP3K1 expression levels between the glioma patients under or over 60 years from TCGA database. The mRNA expression levels of MAP3K1 with different 1p/19q statuses analysed by CGGA batch I (K) and batch II (L). The mRNA expression levels of MAP3K1 with different WHO grades analysed by CGGA batch I (M) and batch II (N). The mRNA expression levels of MAP3K1 with different *IDH* genotypes analysed by CGGA batch I (O) and batch II (P). (Q) MAP3K1 immunoreactivity scores analysed in human para‐tumour and glioma tissues. (R) MAP3K1 immunoreactivity scores analysed in low‐grade and high‐grade glioma tissues. (S) MAP3K1 immunoreactivity scores analysed in grade II, grade III and grade IV glioma tissues. (T) Typical immunohistochemistry images of MAP3K1 in human para‐tumour and different grades of glioma tissues. Scale bar, 50 μm.*p < 0.05, **p < 0.01, ***p < 0.001.

To investigate the potential functions of MAP3K1 in cancer development, we initially examined its expression in different types of human cancer. The results specifically indicated that the expression of MAP3K1 was notably elevated in glioma, which includes both GBM and low‐grade glioma (LGG) (Figure 1A–C). Likewise, MAP3K1 expression was obviously high in 1p/19q non‐codeletion (Figure 1D) and *IDH* wild‐type (Figure 1E) samples. Additionally, the overexpression of MAP3K1 was significantly correlated with age, 1p/19q codeletion, *IDH* status and WHO grade (Table 1). MAP3K1 was found to be highly expressed in high‐grade glioma, especially in GBM, according to the findings from the TCGA dataset (Figure 1F,G). To provide more insight into the clinical significance of MAP3K1 expression in gliomas, we examined the MAP3K1 expression in glioma tissue from patients with varying survival outcomes, such as OS and disease‐free interval (DFI), using data from TCGA. The expression of MAP3K1 was heightened in the dead groups as compared to the living groups (Figure 1H,I). Moreover, the level of MAP3K1 was higher in glioma patients over 60 years old compared with those under 60 years old (Figure 1J). From the CGGA datasets, we proceeded to validate the expression of MAP3K1 in various molecular subtypes and grades of glioma. The expression of MAP3K1 was authenticated by the CGGA datasets (Figure 1K–P). By IHC, we identified the levels of MAP3K1 protein in human glioma and para‐tumour tissues. Increased protein levels of MAP3K1 were found in glioma tissues compared with the para‐tumour tissue (Figure 1Q,T), especially in high‐grade glioma (Figure 1R–T **)**. There was a significant correlation between the protein levels of MAP3K1 and the WHO grade of glioma, as indicated by correlation analysis of MAP3K1 IHC staining with clinicopathologic parameters (Table S1). Collectively, the poor clinicopathological characteristics of glioma were significantly linked to the aberrant expression of MAP3K1. Our results indicate that MAP3K1 might be implicated in glioma progression and function as a promoter in glioma.

**TABLE 1:** Association between MAP3K1 mRNA expression and the clinical parameters of glioma patients in TCGA.

	Low expression of MAP3K1	High expression of MAP3K1	*p*
*n*	348	348	
WHO grade, *n* (%)			
G2	157 (24.7%)	67 (10.6%)	<0.001
G3	100 (15.7%)	143 (22.5%)	
G4	55 (8.7%)	113 (17.8%)	
*IDH* status, *n* (%)			
WT	78 (11.4%)	168 (24.5%)	<0.001
Mut	265 (38.6%)	175 (25.5%)	
1p/19q codeletion, *n* (%)			
Codel	120 (17.4%)	51 (7.4%)	<0.001
Non‐codel	228 (33.1%)	290 (42.1%)	
Age, median (IQR)	43 (33, 56)	48.5 (35.75, 60)	0.003

Abbreviations: IDH, socitrate dehydrogenase, IQR, interquartile range; Mut, mutant; WHO, world health organization; WT, wild type.

### 3.2 MAP3K1 overexpression predicts poor prognosis and is closely related to glioma disease progression

**FIGURE 2:**
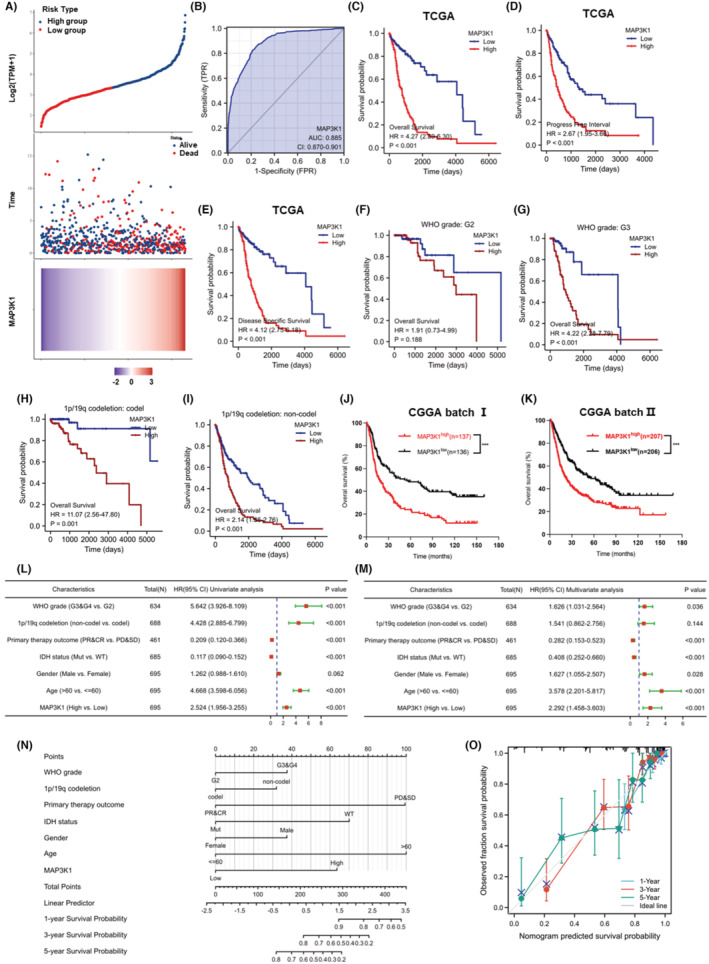
MAP3K1 expression was negatively correlated with the prognosis of glioma. (A) Based on the risk score, patients in TCGA cohorts were divided into high‐ and low‐risk groups. Risk score distribution, survival overview and heatmap of MAP3K1 expression were showed. (B) ROC curve for MAP3K1 in normal tissue and glioma tissues. (C) The overall survival (OS) analysis from TCGA database. The progression free interval (PFI) (D) and disease specific survival (DSS) (E) in TCGA dataset. (F, G) The survival curves comparing the high and low expression of MAP3K1 with different WHO grades from TCGA dataset. (H, I) The survival curves comparing the high and low expression of MAP3K1 with different 1p/19q statuses from TCGA dataset. (J, K) The OS of MAP3K1 expression from the CGGA batch I (J) and batch II (K). Forest plot exhibiting univariate (L) and multivariate regression analysis (M) of MAP3K1 expression and other clinicopathologic parameters in glioma patients. (N) Nomogram for predicting 1‐, 3‐ and 5‐year survival probability of glioma patients in TCGA cohort. (O) Calibration plots of nomogram showing great consistency between predicted and observed 1‐, 3‐ and 5‐year survival probability in TCGA cohort. ***p < 0.001.

We conducted an additional evaluation of the predictive significance of MAP3K1 in glioma. The cohorts were separated into two groups, namely high‐risk and low‐risk, according to the median value of MAP3K1 expression. A higher fatality rate and worse prognosis were found in the MAP3K1 high‐risk group (Figure 2A). Subsequently, we utilized the receiver operating characteristic curve (ROC) to evaluate the ability of MAP3K1 in discerning between glioma samples and normal tissues. The area under the curve (AUC) achieved by MAP3K1 was 0.885, suggesting that MAP3K1 might act as a biomarker and has a good capacity to separate glioma tissues from normal tissues (Figure 2B). In addition, we performed survival analysis including OS, PFI (progression‐free interval) and DSS (disease‐specific survival) and found that the expression of MAP3K1 was negatively associated with the prognosis of glioma (Figure 2C–E). The analysis of glioma patients with high MAP3K1 expression demonstrated poorer prognosis in the grade 3, 1p/19q codeletion and 1p/19q non‐codeletion groups (Figure 2F–I). The OS of MAP3K1 expression was furtherly validated in the CGGA dataset (Figure 2J,K).

Cox regression was used for both univariate and multivariate analyses to determine the prognostic significance of MAP3K1 in the risk signature and clinicopathologic characteristics. As shown in Figure 2L,M, several factors, including WHO grade, primary therapy outcome, age and MAP3K1 expression, were significantly associated with the OS of glioma patients. A nomogram was constructed using Cox regression analysis to predict the survival probability of glioma patients at 1, 3 and 5 years based on MAP3K1 expression and clinicopathologic factors (Figure 2N). Moreover, the predicted results intriguingly matched the actual results of 1‐, 3‐ and 5‐year survival probability in the calibration curves of the nomogram (Figure 2O). All results indicate that the overexpression of MAP3K1 holds great potential as a diagnostic and prognostic biomarker for glioma and is significantly linked to the progression of the disease.

### 3.3 Analysis of immune infiltration and functional enrichment of MAP3K1 in Glioma

To explore the relationship between MAP3K1 and immune infiltration in glioma, we began by evaluating how MAP3K1 expression correlates with the presence of various immune cell types. Our analysis revealed a positive association between MAP3K1 expression and the enrichment of Th2 cells, T helper cells, macrophages, eosinophils, activated dendritic cells (aDCs), and neutrophils within the glioma microenvironment.; MAP3K1 had strong negative correlations with CD8^+^ T cells, T follicular helper (TFH) cells, DCs, mast cells, regulatory T cells (Tregs), pDCs, and natural killer (NK) CD56^bright^ cells in glioma (Figure S1A,B). Furthermore, we investigated immune infiltration levels in glioblastoma (GBM) associated with various somatic copy number alterations (SCNAs) of MAP3K1. Compared to normal tissue, glioma samples exhibiting MAP3K1 arm‐level deletions showed significantly increased infiltration of CD4^+^ T cells, neutrophils, and dendritic cells. (Figure S1C).

### 3.4 Analysis of functional enrichment related to MAP3K1 in glioma

**FIGURE 3:**
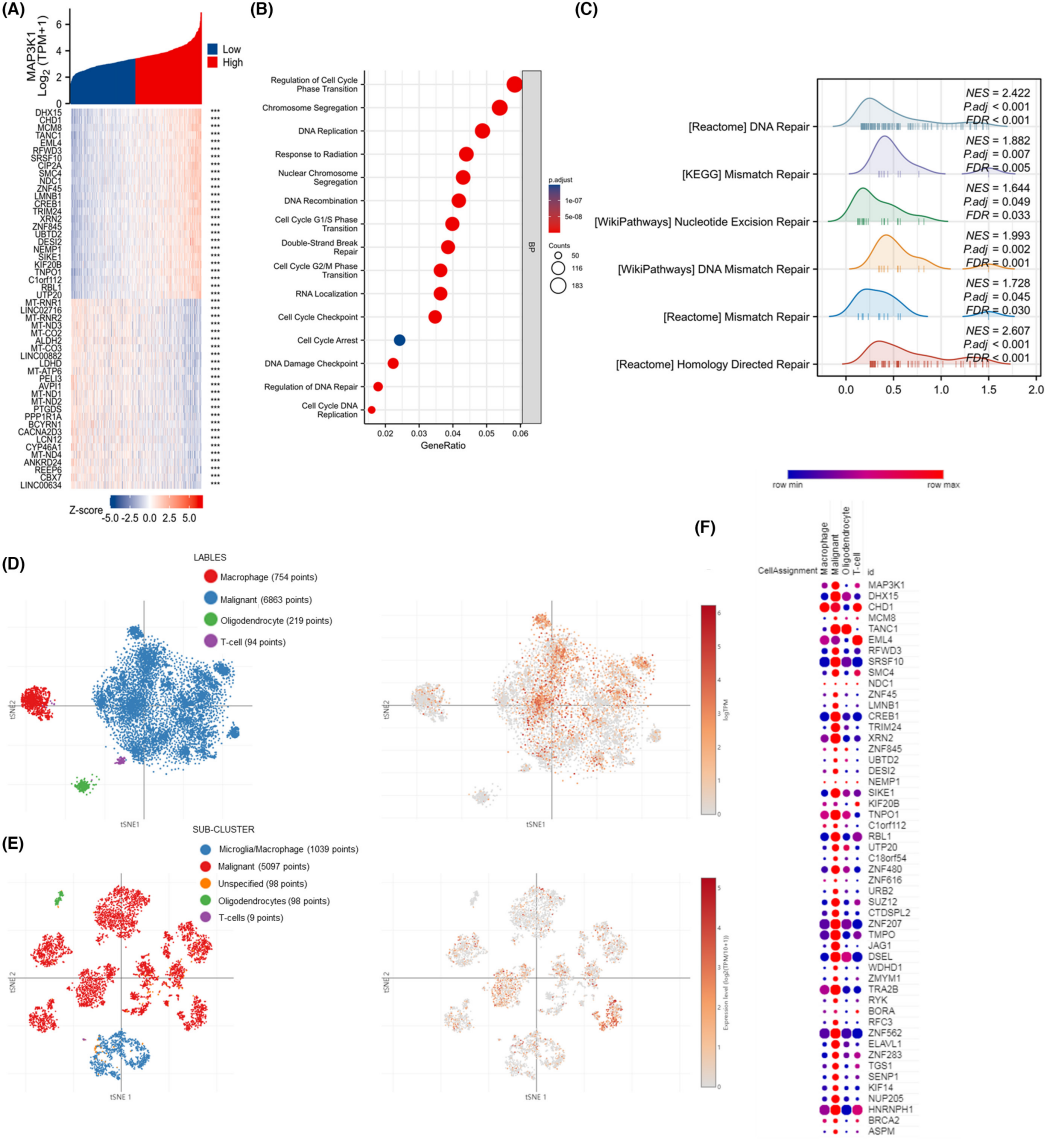
Function enrichment analysis of MAP3K1 in glioma. (A) The heatmap of the top 25 genes most positively or negatively correlated with MAP3K1. (B) GO enrichment analysis for biological process (BP) of MAP3K1 in TCGA dataset. (C) GSEA enrichment analysis of MAPK3K1 and its co‐expression genes in glioma by TCGA data was performed. The t‐SNE of MAP3K1 expression in different sub‐clusters of gliomas from study 1 (D) and study 2 (E). (F)The top 25 genes positively associated with MAP3K1 in glioma from TCGA mainly enriched in malignant tumor cells along with macrophage cells.

To elucidate the role of MAP3K1 in glioma, we analysed genes that are co‐expressed with MAP3K1 in the TCGA dataset. This analysis identified the top co‐expressed genes, highlighting those with either positive or negative correlations with MAP3K1 (Figure 3A). Gene ontology (GO) enrichment analysis showed that the BP of MAP3K1 co‐expressed genes were mainly enriched in the regulation of cell cycle phase transition, DNA replication, DNA recombination, double‐strand break repair, cell cycle checkpoint, cell cycle arrest, DNA damage checkpoint and regulation of DNA repair (Figure 3B). GSEA analysis showed that DNA repair, homology directed repair, DNA mismatch and repair pathways were enriched in gliomas with high MAP3K1 expression (Figure 3C). Using single‐cell RNA sequencing datasets, we performed t‐SNE analysis to determine the expression of MAP3K1 in glioma. The results suggested that MAP3K1 was enriched in malignant cells along with macrophages (Figure 3D,E). Coincidentally, the top 25 genes positively correlated with MAP3K1 in the TCGA dataset were predominantly enriched in glioma cells (Figure 3F). These results indicated that MAP3K1 was mainly expressed in glioma cells and might be involved in the regulation of the DNA repair.

### 3.5 MAP3K1 silencing suppresses the migration and has no effect on the proliferation of GBM cells

**FIGURE 4:**
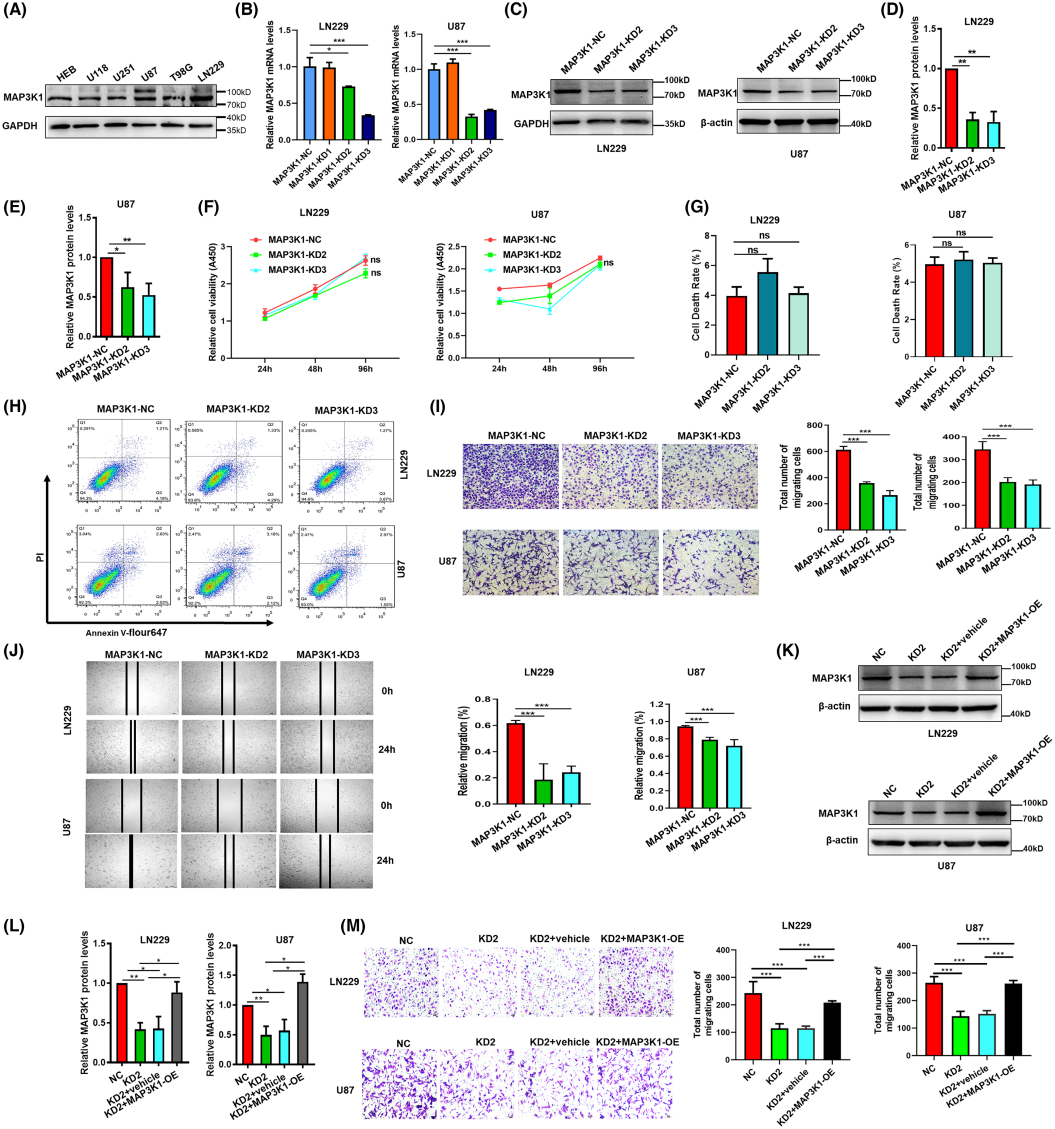
MAP3K1 Silencing suppresses the migration and had no effect on the proliferation of GBM cells. (A) Western blot were performed to analyse the expression of MAP3K1 in GBM cell lines and HEB. The cells were transfected with different MAP3K1‐siRNA (MAP3K1‐KD1, MAP3K1‐KD2 and MAP3K1‐KD3) and scramble sequence siRNA (MAP3K1‐NC). MAP3K1 was silenced by siRNA in U87 and LN229 cells. (B) Knockdown efficiency was evaluated by RT‐qPCR. (C–E) Knockdown efficiency was evaluated by western blot. Protein intensity was analysed by densitometry and the levels were normalized to β‐actin in U87 or GAPDH in LN229. (F) The viability of U87 and LN229 cells under MAP3K1 knockdown was evaluated by CCK8. Flow Cytometry analysed the cell death (G, H) of MAP3K1 knockdown in U87 and LN229 cells. (I) Transwell assay analysed the migration ability of U87 and LN229 cells under MAP3K1 knockdown. (J) Wound scratch assay analysed the migration ability of U87 and LN229 cells under MAP3K1 knockdown. (K, L) MAP3K1 was overexpressed in MAP3K1 silenced GBM cells. The cells were transfected with siRNA MAP3K1‐KD2 (KD2) and scramble sequence siRNA (NC). The MAP3K1 silenced GBM cells were transfected pcDNA3.1 (vehicle) or pcDNA3.1‐ MAP3K1 (MAP3K1‐OE). Western blot was performed to analyse the expression of MAP3K1 in U87 and LN229 cells. (M) Transwell assay analysed the migration ability of U87 and LN229 cells. Data represent three independent experiments. *p < 0.05, **p < 0.01, ***p < 0.001.

To study the biological functions of MAP3K1 in glioma, the expression of MAP3K1 in GBM cells was knocked down. We evaluated the protein levels of MAP3K1 in GBM cell lines and selected LN229 and U87 cells for subsequent experiments because they exhibited higher levels of MAP3K1 protein expression. (Figure 4A). MAP3K1 was significantly knocked down in LN229 and U87 cells (Figure 4B–E). MAP3K1 knockdown had no effect on the proliferation of GBM cells in vitro after 24, 48, and 96 h (Figure 4F). No significant changes in GBM cells death were observed by MAP3K1 knockdown (Figure 4G,H). Furthermore, the inhibition of MAP3K1 resulted in a significant decrease in the migration of LN229 and U87 cells (Figure 4I,J). MAP3K1 was overexpressed in MAP3K1 silenced GBM cells for rescue experiment (Figure 4K,L). As shown in the Figure 4M, MAP3K1 was overexpressed and MAP3K1 overexpression reversed the migration inhibition induced by MAP3K1 silencing in U87 and LN229 cells.

### 3.6 MAP3K1 silencing enhances the TMZ sensitivity of GBM

**FIGURE 5:**
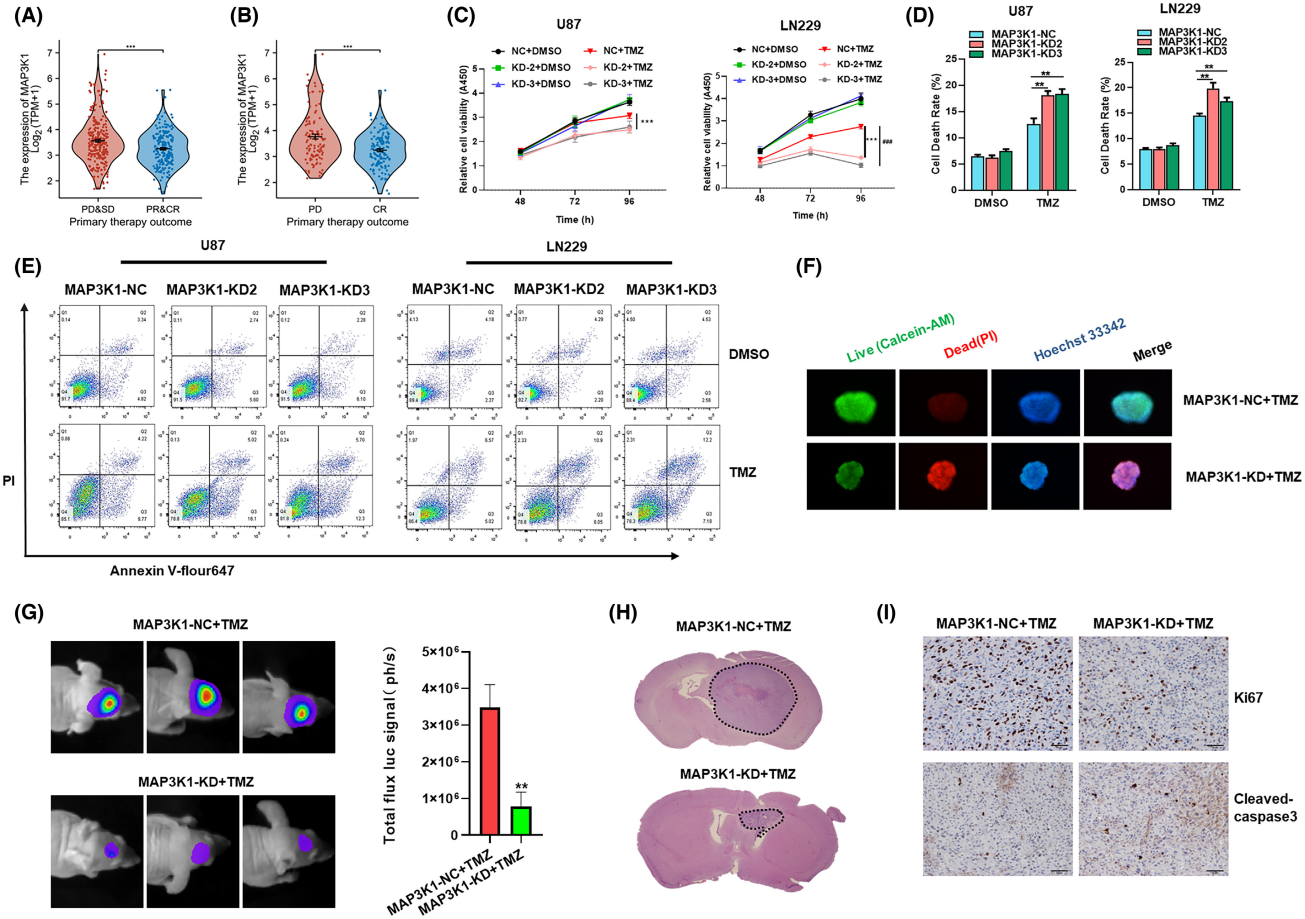
MAP3K1 silencing enhances the TMZ sensitivity of GBM. (A, B) The expression of MAP3K1 in different primary therapy outcome of glioma patients in TCGA database. PD, progressive disease; SD, stable disease; PR, partial response; CR, complete response. (C) The viability of MAP3K1 silenced U87 and LN229 cells under TMZ treatment was evaluated by CCK8. (D, E) Flow Cytometry analysed the cell death of MAP3K1 knockdown U87 and LN229 cells under TMZ (200 μM) treatment for 72 h. (F) MAP3K1 silenced GBM patients‐derived organoids were treated by TMZ, the growth and cell death of GBM patients‐derived organoids were detected by 3D live dead cell viability assay. (G) Representative bioluminescent images and the quantification of the U87 tumour‐bearing mice on Days 21. The data are shown as mean ± SD (*n* = 5). (H) The growth of the glioma from tumour‐bearing mice was evaluated by HE staining method. (I) Representative IHC images of the intracranial glioma tissue from tumour‐bearing mice stained by Ki67 and cleaved‐caspase3. **p < 0.01, ***p < 0.001, ###*p* < 0.001.

**FIGURE 6:**
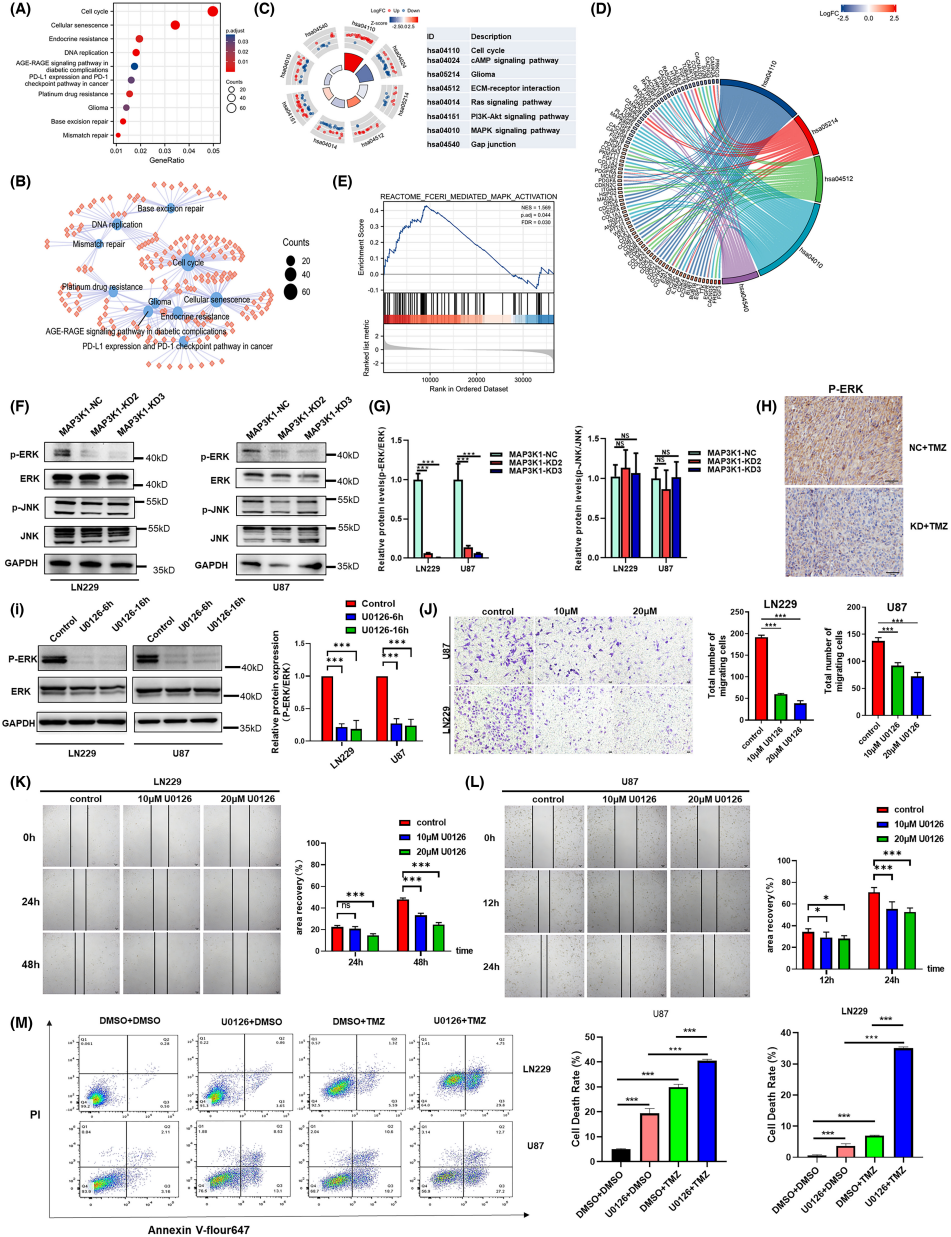
MAP3K1 could potentially enhance the migration and TMZ resistance of GBM cells via MEK/ERK pathway. (A) KEGG pathway analysis of MAP3K1 in glioma. (B) The enriched KEGG pathway terms of MAP3K1 and its co‐expression genes in glioma. (C) Circle plot of enriched KEGG pathway. The bar plots are shown in the inner ring which the height of the bar indicates the significance of the term, and colour corresponds to the z‐score. Expression levels (logFC) for the genes in each term are shown in the scatter plots of outer ring. (D) Chord plot of enriched KEGG pathway; the genes are linked via ribbons to their assigned terms. (E) GSEA of MAP3K1 and its co‐expression genes in glioma. (F, G) Western blot analysed the level of ERK, p‐ERK, JNK and p‐JNK in the MAP3K1 silenced GBM cells. (H) Representative IHC images of the intracranial glioma tissue from tumour‐bearing mice stained by p‐ERK. (I) U87 and LN229 cells were treated with U0126 (10 uM) for 6 and16 h. Western blot analysed the level of ERK and p‐ERK in U87 and LN229 cells. (J) Transwell assay analysed the migration ability of U87 and LN229 cells under the treatment with U0126. (K, L) Wound scratch assay analysed the migration ability of U87 and LN229 cells under the treatment with U0126. (M) Flow Cytometry analysed the cell death of U87 and LN229 cells under indicated treatment for 72 h. TMZ (200 μM) and U0126 (10 uM) were used. Data represent three independent experiments. *p < 0.05, ***p < 0.001.

With the progress of chemotherapy, numbers of patients encounter the problem of TMZ resistance, which leads to the failure of treatment. The phenomenon of drug resistance is widespread and increasingly prominent, which greatly limits the clinical efficacy of TMZ.^7^, ^8^, ^9^ The analysis performed on samples from the TCGA dataset showed that expression of MAP3K1 mRNA level was enriched in the progressive disease (PD) and stable disease (SD) group compared with the complete response (CR) and partial response (PR) group of primary therapy outcomes (Figure 5A). MAP3K1 mRNA level was also enriched in the PD group compared with the CR group (Figure 5B). As shown in Figure 3C, pathways including DNA repair, homology directed repair and DNA mismatch repair pathways were enriched in gliomas with high MAP3K1 expression. KEGG pathway analysis demonstrated that mismatch repair and platinum drug resistance were enriched in gliomas with high MAP3K1 expression (Figure 6A). We speculate that MAP3K1 may be involved in TMZ sensitivity of GBM. MAP3K1 knockdown exacerbated the TMZ induced inhibition of glioma cell proliferation (Figure 5C) and increased the cell death of GBM cells induced by TMZ (Figure 5D,E).

To further verify the effect of MAP3K1 on TMZ sensitivity of GBM cells, we established GBM patients‐derived organoids. The expression of MAP3K1 in GBM patients‐derived organoids was knockdown by lentivirus infection. After 3 weeks of TMZ treatment, the growth and cell death of GBM patients‐derived organoids were detected by 3D survival staining fluorescence immediately. The results showed that MAP3K1 knockdown combined with TMZ treatment significantly inhibited the growth and increased cell death of GBM patients‐derived organoids compared to the control group (Figure 5F). The results showed that MAP3K1 knockdown significantly increased the TMZ sensitivity of GBM derived organoids to TMZ.

To further confirm the role of MAP3K1 in vivo, intracranial glioma model of nude mice was constructed by using negative control and MAP3K1 stable knockdown cell lines. TMZ were injected into each group, and tumour growth was monitored weekly by in vivo using bioluminescent imaging. After TMZ injection, the tumour volume of MAP3K1 knockdown group was significantly reduced compared with the control group (Figure 5G). Whole brain HE staining showed that tumours in MAP3K1 knockdown group were smaller and more defined (Figure 5H). Immunohistochemical analysis showed that MAP3K1 knockdown combined with TMZ decreased the positive rate of Ki‐67 and increased the expression of cleaved‐caspase3 (Figure 5I). MAP3K1 knockdown can enhance TMZ‐induced proliferation inhibition and death of glioma cells. Those results were consistent with our experiments in vitro and indicated that MAP3K1 knockdown reversed TMZ resistance in GBM.

### 3.7 MAP3K1 could enhance the migration and TMZ resistance of GBM cells via MEK/ERK pathway

We conducted pathway enrichment analysis of MAP3K1 in glioma to investigate its potential molecular mechanism. The results of KEGG pathway analysis demonstrated that pathways, including the cell cycle, DNA replication, cellular senescence, mismatch repair, platinum drug resistance, glioma, PD‐L1 expression and the PD‐1 checkpoint pathway in cancer were enriched in gliomas with high MAP3K1 expression (Figure 6A). The relationship between the KEGG pathway and the molecule is shown in the KEGG enrichment network (Figure 6B). KEGG pathway analysis in conjunction with logFC showed that the cell cycle, glioma, ECM‐receptor interaction, and MAPK signalling pathway might be regulated by MAP3K1 in glioma (Figure 6C,D). In addition, GSEA showed that FceRI‐mediated MAPK activation was enriched (Figure 6E). Then, we focused on the MAPK signalling pathway and performed western blot assay. We found that the phosphorylation level of ERK was significantly decreased by MAP3K1 silencing. No significant change in the JNK pathway was found in MAP3K1‐silenced U87 and LN229 cells (Figure 6F,G). We analysed the protein level of p‐ERK in intracranial glioma tissue from tumour‐bearing mice by Immunohistochemical. Immunohistochemical result showed that MAP3K1 knockdown combined with TMZ decreased the protein level of p‐ERK in intracranial glioma tissue (Figure 6H).

We also explored the role of ERK in GBM cell migration. The treatment of MEK inhibitor (U0126) caused a significant inhibition of ERK phosphorylation (Figure 6I) and resulted in a significant inhibition of migration of LN229 and U87 cells which were detected by transwell chamber and scratching assay (Figure 6J–L). In addition, the U0126 treatment significantly enhanced the death induced by TMZ in U87 and LN229 cells (Figure 6M). Based on our findings, it can be inferred that MAP3K1 potentially enhances the migration and TMZ resistance of GBM cells through the MEK/ERK pathway.

## 4 DISCUSSION

Currently, glioma is still malignant and notorious due to its elevated morbidity, recurrence rate, mortality, and low success rate in finding a cure. The clinical outcomes of glioma are far from satisfactory using current treatments.^5^ There are very few treatment options available for glioma patients at present, and it is of utmost importance to find new targets for therapeutic intervention. Our study demonstrates that MAP3K1 silencing suppresses the migration of GBM cells, facilitates the TMZ sensitivity and potentially plays a role in anti‐tumour immune regulation. MAP3K1 might functions as a robust therapeutic target for GBM.

To enhance the precision of grade glioma classification and to facilitate the selection of appropriate therapies, there is a pressing need for more precise, efficient and accurate diagnostic and prognostic biomarkers in glioma. We generated a ROC and performed survival analysis and univariate and multivariate analyses. A nomogram was built to predict the probability of glioma patients' survival at 1, 3 and 5 years. Our findings indicate that the overexpression of MAP3K1 serves as a promising diagnostic and prognostic biomarker for glioma. Even though multiple datasets we performed have analysed the role of MAP3K1 in diagnosing and predicting the outcome of glioma, further research is required to conclusively establish MAP3K1 as a new diagnostic marker and prognostic indicator for glioma.

To date, increasing evidence has indicated that MAP3K1 is involved in the progression of cancers. For instance, MAP3K1 functions as a causative gene among several susceptibility alleles^16^ and promotes breast cancer cell survival.^22^ MAP3K1 is highly expressed in pancreatic cancer patients,^19^ and the metastasis of human pancreatic cancer cells is suppressed by a reduction in MAP3K1 activity.^18^, ^19^ However, the expression of activated MEKK1 induces apoptosis in prostate cancer cells in a manner dependent on the presence of androgen receptor.^23^ Previous findings have shown that melanocyte‐specific deletion of MAP3K1 accelerates melanomagenesis in mice.^17^ At present, the role of MAP3K1 in cancer cell proliferation and death is still controversial. In our study, knockdown of MAP3K1 had no effect on the proliferation and cell death of U87 and LN229 cells. For cell migration, knockdown of MAP3K1 inhibited the migration of embryonic stem cells in the Boyden chamber chemotaxis assay.^22^ In epidermal keratinocytes, the migration induced by TGF‐β, activin A and activin B is defective in MAP3K1‐KD mice.^13^ Similar results were obtained in glioma cells in which MAP3K1 silencing suppressed the migration of GBM cells. More work is needed to fully elucidate the role of MAP3K1 in glioma.

As a first‐line chemotherapy drug for high‐grade glioma, TMZ resistance is closely related to the failure of GBM treatment. Mechanisms like repair of O6‐methylguanine‐DNA methyltransferase (MGMT), activation of DNA damage repair system, emergence of stem cells and acquired resistance are involved in TMZ resistance.^24^ In present study, pathways including DNA repair, homology directed repair, platinum drug resistance, DNA mismatch and repair pathways were enriched in gliomas with high MAP3K1 expression. We speculate that MAP3K1 may be involved in TMZ sensitivity of GBM by activation of DNA damage repair system.

Searching for new targets for GBM treatment and developing new drugs to reverse TMZ resistance have important clinical implications for reducing the incidence, recurrence, and mortality of glioblastoma. Here, we found that MAP3K1 knockdown reversed TMZ resistance of GBM by in vivo and in vitro experiments. Using GBM patients‐derived organoids, we found that MAP3K1 knockdown combined with TMZ treatment significantly enhanced the TMZ sensitivity. Jia Wang et al. demonstrated that increased expression of TRIB2 and MAP3K1 were associated with resistance to TMZ and radiotherapy by bioinformatics analysis.^5^ Their results were consistent with our bioinformatics analysis. Additionally, we further verify the effect of MAP3K1 on TMZ sensitivity of GBM cells in vivo and in vitro. Meanwhile, we initially revealed the molecular mechanism that MAP3K1 enhances TMZ resistance of GBM cells through the MEK/ERK pathway.

## 5 CONCLUSIONS

MAP3K1 serves as a reliable diagnostic and prognostic biomarker for glioma. MAP3K1 could potentially facilitate the migration and TMZ resistance of GBM cells through MEK/ERK signalling. MAP3K1 was correlated with the immune infiltration in glioma. MAP3K1 may play a likely role in regulating the antitumor immune response to promote the progression of glioma. The inhibition of MAP3K1 may be a promising approach to overcome TMZ resistance and improve outcomes of GBM patients.

## AUTHOR CONTRIBUTIONS

**Sicheng Wu:** Data curation (equal); formal analysis (equal); investigation (equal); writing – original draft (equal). **Senrui Xue:** Methodology (equal); software (equal). **Yuchen Tang:** Data curation (equal). **Wenyu Zhao:** Supervision (equal). **Maojin Zheng:** Resources (equal). **Zhixuan Cheng:** Methodology (equal). **Xin Hu:** Visualization (equal). **Jinmin Sun:** Resources (equal); validation (equal); writing – review and editing (equal). **Jing Ren:** Conceptualization (equal); funding acquisition (equal); investigation (equal); writing – review and editing (equal).

## CONFLICT OF INTEREST STATEMENT

The authors declare that they have no competing interests.

## Supporting information


**Data S1:** Supporting Information.

## Data Availability

The datasets supporting the conclusions of this article are available in the TCGA repository(https://www.cancer.gov/about‐nci/organization/ccg/research/ structural‐ genomics/tcga), CGGA repository (http://www.cgga.org.cn/), GTEx repository (https://www.genome.gov/Funded‐Programs‐Projects/Genotype‐Tissue‐Expression‐Project). Other data in current study are available from the corresponding author on reasonable request.
