# Supplementary material for: Mitogen‐activated protein kinase kinase kinase 1 facilitates the temozolomide resistance and migration of GBM via the MEK/ERK signalling
Source: J Cell Mol Med. 2024 Oct 23;28(20):e70173. doi: 10.1111/jcmm.70173 (PMC11499072; doi:10.1111/jcmm.70173)
Supplement: Supplementary file 1 — Data S1: Supporting Information. [file JCMM-28-e70173-s001.docx]

**Supplementary Materials**

**Supplementary Figure1**

**
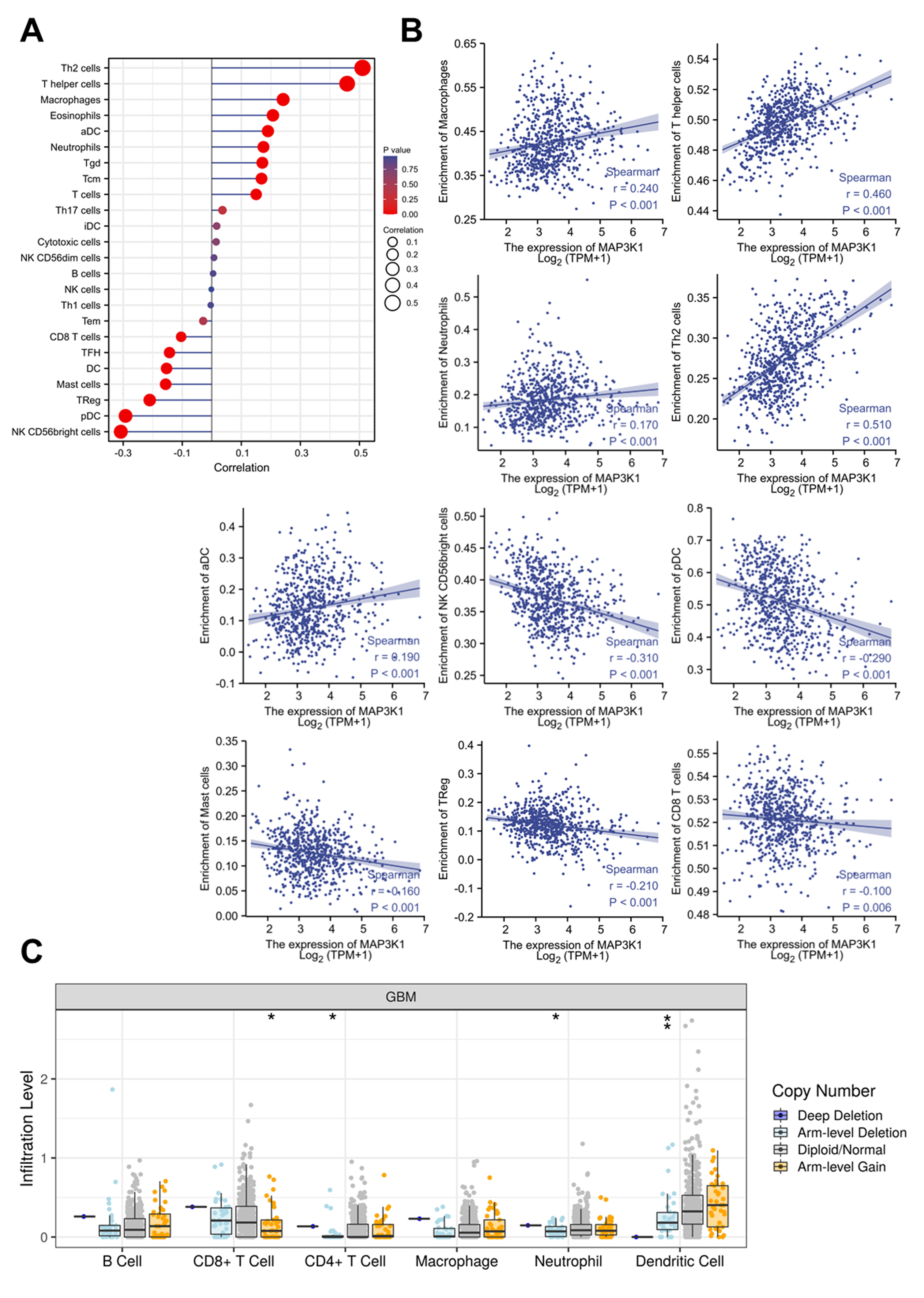
**

**sFIGURE 1** |Correlation and association analysis between MAP3K1 and immune infiltration in glioma. (A-B) Correlation analysis between immune cells enrichment and MAP3K1 expression in glioma. The infiltration levels of immune cells were quantified by ssGSEA analyzed by spearman correlation. (C) The infiltration levels of various immune-related cells under different SCNAs of MAP3K1 in glioma.

**Table**

**Supplementary Table 1:** MAP3K1 IHC staining and clinicopathological characteristics of 96 glioma patients

| Variable | Number（n） | MAP3K1 staining | | | |
| --- | --- | --- | --- | --- | --- |
|  |  | **Low (%)** | **High (%)** | ***χ^2^*** | ***p*-value** |
| gender | - | - | - | 0.009 | 0.923 |
| Male | 59 | 42(71.2) | 17(28.8) | - | - |
| Female | 37 | 26(70.3) | 11(29.7) | - | - |
| Age (years) | - | - | - | 0.707 | 0.141 |
| <50 | 44 | 32(72.7) | 12(27.3) | - | - |
| ≥50 | 52 | 36(69.2) | 16(30.8) | - | - |
| Tumor size (cm) | - | - | - | 2.692 | 0.101 |
| <5 | 31 | 26(83.9) | 5(16.1) | - | - |
| ≥5 | 29 | 19(65.5) | 10(34.5) | - | - |
| WHO grade | - | - | - | 5.928 | 0.015 |
| Low (Ⅰ-Ⅱ) | 27 | 24(88.9) | 3(11.1) | - | - |
| High (Ⅲ-Ⅳ) | 69 | 44(66.8) | 25(36.2) | - | - |

**Methods**

**1.1. Univariate and Multivariate Cox Analyses**

R package "survival" was used for univariate and multivariate Cox analyses including MAP3K1 expression, WHO grade, 1p/19q codeletion, *IDH* status, primary therapy outcome, gender, and age.

**1.2. Constitution of a Risk Model**

We performed multivariate Cox proportional hazards regression analyses and determined risk scores based on the Cox coefficients. A risk score formula of the expression levels and coefficients of genes was performed.

The application of Cox regression analyses aimed to find the genes that had a significant relationship with the overall survival (OS) in the TCGA database and create a risk signature. Each patient's individual risk score was calculated using the formula: risk score = Ʃ(βi × Expi), where i represented the number of prognostic genes, β was the regression coefficient value for each gene, and Exp denoted the gene expression level. Based on the median risk score, the patients were divided into a high-risk group and a low-risk group.

**1.3. Analyses of the Relationships between MAP3K1** **Expression and Prognosis, Diagnosis, and Clinical Phenotype**

The Kaplan–Meier method and Cox regression were used to investigate the relationship between MAP3K1 expression and patient prognosis. In addition, the R packages “survival” and “survminer” were utilized to analyze survival curves and produce the plots. To assess the diagnostic significance of MAP3K1, receiver operating characteristiccurves (ROC) was utilized, along with the R packages "pROC" and "ggplot2".

**1.4. Analyses of** **Function and Pathway Enrichment**

We used Pearson’s correlation coefficients (|r| > 0.4, p < 0.001) to screen for relevant genes that were co-expressed with MAP3K1 in the TCGA database. To investigate the biological functions and signaling pathways linked to MAP3K1, the R package "clusterProfiler" was utilized for Gene Ontology (GO) and Kyoto Encyclopedia of Genes and Genomes (KEGG) enrichment analyses. We performed gene set enrichment analysis (GSEA) using the R package "clusterProfiler". We deemed |NES| > 1, p < 0.05, and false discovery rate (FDR) < 0.25 as indicators of statistical significance.

**1.5. Single-Cell RNA Sequencing Analyses**

Data from single-cell RNA-seq was gathered from the Broad Institute Single-Cell Portal in study 1 (https://singlecell.broadinstitute.org/single_cell/study/SCP393/ single-cell-rna-seq-of-adult-and-pediatric-glioblastoma#study-download), and study 2 (<https://singlecell.broadinstitute.org/single_cell/study/SCP50/single-cell-rna-seq-analysis-of-astrocytoma>).

**1.6. Relationships between MAP3K1 and Immunity**

We used the R package "GSVA" to quantify the levels of immune cell infiltration in glioma. We utilized the single-sample gene set enrichment analysis (ssGSEA) algorithms integrated in this package. We constructed the somatic copy number alteration (SCNA) module using the web server TIMER (https://cistrome.shinyapps.io/timer/).
